# Compounded medications for cardiovascular use in neonatology: an integrative review

**DOI:** 10.1590/1984-0462/2023/41/2021167

**Published:** 2022-09-09

**Authors:** Lucas Louro Greenhalgh, Márcia Maria Barros dos Passos, Arthur Lopes Agrizzi, Mariana Sato de Souza Bustamante Monteiro

**Affiliations:** a Universidade Federal do Rio de Janeiro, Rio de Janeiro, RJ, Brazil.

**Keywords:** Drugs utilization, Infant, newborn, Pharmaceutical preparations, Off-label use, Intensive care units, neonatal, Uso de medicamentos, Recém-nascido, Preparações farmacêuticas, Uso off-label, Unidades de terapia intensiva neonatal

## Abstract

**Objective:**

To analyze the profile of the compounded cardiovascular medicines prescribed in neonatology in Brazil.

**Data source:**

An integrative bibliographic review was carried out, including studies published in the last 20 years. The used descriptors were: *Intensive Care Neonatal, Off-Label Use, Pharmaceutical Preparations*, in the databases Virtual Health Library (VHL), PubMed, and Scientific Electronic Library Online (SciELO). Review articles and guidelines were excluded. The quality of the evidence was analyzed, and 10 articles were selected to integrate the study.

**Data synthesis:**

The profile of routine prescrption in the neonatal unit was evaluated. The main cardiovascular medications prescribed as compounding formulation were: Spironolactone, Captopril, Furosemide, Hydrochlorothiazide, Propranolol, Amiodarone, Nifedipine, Carvedilol, Digoxin, Enalapril, Epinephrine, and Hydralazine. The drugs were obtained from adaptations of dosage forms, through the transformation of capsules or tablets into liquid formulations, as a solution, suspension, or syrup, as well as in the form of solutions prepared from active pharmaceutical ingredients. The compounding of medications made drug therapy possible in neonatology, considering that such medications do not have registration of the oral liquid dosage form in the country, despite being part of the List of Essential Medicines for Children of the Word Health Organization.

**Conclusions:**

It was possible to analyze the profile of compounded cardiovascular medicines prescribed in neonatology in Brazil. The results showed the need for the development of medications suitable for the neonatal population, and the standardization of operational procedures for preparing extemporaneous formulations in neonatology to increase drug safety.

## RESUMO

**Objetivo:**

Analisar o perfil dos medicamentos cardiovasculares manipulados prescritos em neonatologia no Brasil.

**Fontes de dados:**

Foi realizada uma revisão bibliográfica integrativa, incluindo estudos publicados nos últimos 20 anos. As palavras-chave utilizadas foram: *intensive care neonatal, off-label use, pharmaceutical preparations*, nos bancos de dados da Biblioteca Virtual em Saúde (BVS), PubMed e Biblioteca Eletrônica Científica Online (SciELO). Os artigos de revisão e diretrizes foram excluídos. A qualidade da evidência foi analisada, e foram selecionados 10 artigos para integrar o estudo.

**Síntese dos dados:**

Todos os estudos possibilitaram a avaliação do perfil de prescrição na rotina da unidade. Os principais fármacos cardiovasculares, prescritos em formulação extemporânea, foram espironolactona, captopril, furosemida, hidroclorotiazida, propranolol, amiodarona, nifedipina, carvedilol, digoxina, enalapril, epinefrina e hidralazina. Os medicamentos foram obtidos mediante adaptações de formas farmacêuticas, por meio da transformação de cápsulas ou comprimidos em formulações líquidas, como solução, suspensão ou xarope, assim como também na forma de soluções preparadas com base nos insumos farmacêuticos ativos. A manipulação de medicamentos tornou possível o tratamento medicamentoso em neonatologia, uma vez que tais medicamentos não possuem registro da forma farmacêutica líquida oral no país, apesar de integrarem a lista de medicamentos essenciais em pediatria da Organização Mundial da Saúde.

**Conclusões:**

Foi possível analisar o perfil dos medicamentos cardiovasculares manipulados prescritos na neonatologia no Brasil. Os resultados evidenciaram a necessidade do desenvolvimento de medicamentos adequados à população neonatal e à padronização dos procedimentos operacionais do preparo de formulações extemporâneas na neonatologia, para aumentar a segurança dos medicamentos.

## INTRODUCTION

The pediatric age group is known to be a therapeutic orphan, due to the low availability of formulations adapted to this population. As a result, there is a high prevalence of off-label and/or unlicensed use of medications.^1–5^ Turner^6^ proposes the classification of off-label medication use when the medication is used in the dose, age, route of administration, and/or indication other than registration, while its unlicensed use is defined by the use of extemporaneous formulations.^7^

Newborns or neonates (0–28 days after birth) use off-label drugs 90% of the time and can reach 100% in neonatal intensive care units. Due to the lack of information on the safety, efficacy, and quality of these drugs, a high frequency of adverse drug events (ADE) is observed in this population, especially among hospitalized patients.^8–11^

Several studies^8,12–14^ have shown that extemporaneous formulations are widely used, due to the lack of liquid dosage forms for oral use suitable for neonatology, especially for medicines of the cardiovascular pharmacological group. This is due to the patient’s physiological and anatomical immaturity.^2,4^ In this context, formulations for oral use are the ones with the best cost-effectiveness and lower risk, allowing better adherence, compatibility with medicine dispensers, and greater ease of administration (which can be done by tubes); however, some disadvantages have been reported, such as the difficulty in guaranteeing stability, the palatability of the formulation, the possibility of interaction with the tube and with formulas that replace milk and with other medications.^9–16^ Such factors must be evaluated, standardized, and validated during the preparation of formulations in the hospital setting, including the addition of excipients to maintain the homogeneity and stability of the pharmaceutical formulation.^17^

It is worth considering that drugs registered in the oral liquid dosage form can be good alternatives for use in the practice scenario, after evaluating their specificities and their suitability for the neonatal population, even with regard to the toxicity of the excipients and the dose.^3,17–19^

In this scenario, in line with the document *Pediatric pharmaceutical services in Brazil: recommendations and strategies to expanding coverage, access and rational use of medicines in children*,^20^ the present review aimed to map the cardiovascular medicines used in neonatology in Brazil, considering those that have unlicensed use, related to the transformation of the original dosage form, described in the literature, but which are not registered in the Brazilian Health Regulatory Agency (*Agência Nacional de Vigilância Sanitária* – ANVISA). With this mapping, the authors proposed that safe alternatives be sought for the use of these drugs, guiding both the facilitated registration based on formulations already registered in other countries and the validation, standardization, and provision of information on methods for compounding extemporaneous formulations in neonatology.

## METHOD

An integrative review of the literature was carried out, through the established guiding question: what are the cardiovascular compounded medications needed in neonatology inpatient units in Brazil? To answer this question, searches were performed using the descriptors *intensive care, neonatal, off-label use*, and *pharmaceutical preparations*. The consulted databases were the Virtual Health Library (VHL), PubMed, and the Scientific Electronic Library Online (SciELO). The search was individually carried out by two researchers in June and July 2020 and considered publications from the last 20 years. After reading the abstracts, according to relevance and consistency, the following inclusion criteria were applied: original articles written in Portuguese, Spanish, and English languages; open access articles; and focused on Brazil. Exclusion criteria were: opinion articles; reflection articles; congress proceedings; editorials; articles that do not directly address the topic or that did not include neonatal patients in the scope of the study.

The results were compared with documents of the British National Formulary for Children (BNFC)^21^ to assess information on the registration of the oral liquid dosage form in the United Kingdom, as well as to verify if the medicines were part of the List of Essential Medicines for Children of the World Health Organization,^22^ if the medications had a registration of the oral liquid dosage form at ANVISA, through the System of Consultation to Registered Products (*Sistema de Consulta a Produtos Registrados*)^23^, and if there was information on the use of these medicines in the National Therapeutic Form (*Formulário Terapêutico Nacional*) for neonatal patients.^24^

## RESULTS

**Figure 1:**
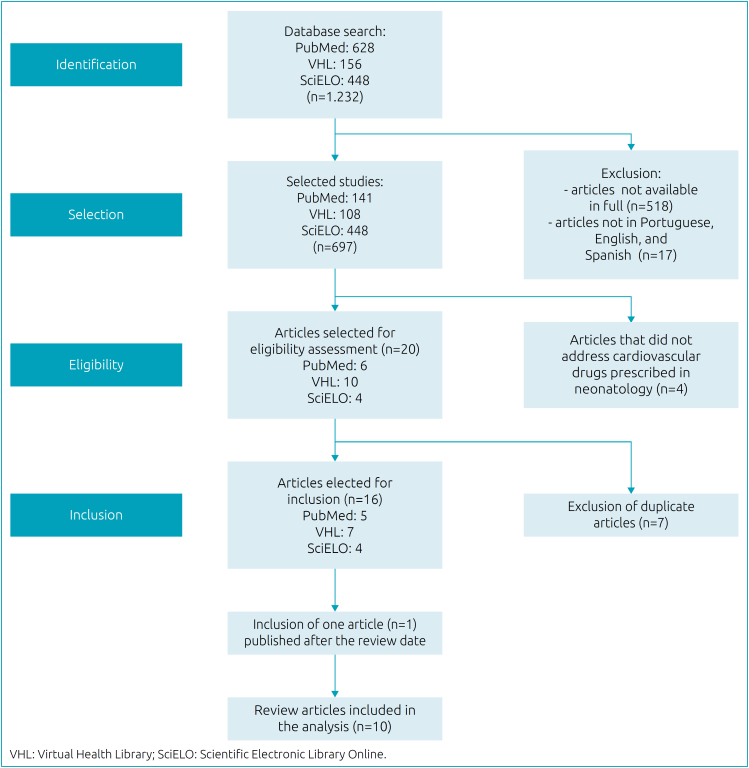
Flowchart of articles’ selection for the integrative review.

A total of 16 articles were chosen, which addressed cardiovascular medications prescribed in neonatology. Seven duplicate articles were excluded and one from an alternative source was added. The 10 final articles were included for evaluation and discussion. The flowchart of the process for selecting articles for analysis is shown in Figure 1.

The 10 studies were named by the editors as originals. As for the types of study, there were five descriptive studies; two cross-sectional studies; two cohort studies; and one prospective study, covering the years of publication between 2009 and 2020. All studies enabled to evaluate the prescription profile within the routine of the unit. All studies described the use of cardiovascular medications compounded for neonatology in a Neonatal Intensive Care Unit (NICU), according to the inclusion criteria.

The 10 articles used in the analysis of the present study are described in Table 1, ordered by author, publication date, objectives, and results, containing the description of the cardiovascular medicines mentioned in each study.

**Table 1:** General characteristics of included studies on cardiovascular medicines for use in neonates as an extemporaneous formulation.

Study	Study design	Objectives	Mentions of cardiovascular medicines
Passos et al., 2020^25^	Retrospective descriptive study	To describe the prescriptions of compounded medications for neonate patients and verify their pharmacotechnical preparation	NICU: Spironolactone, Captopril, Furosemide, Hydrochlorothiazide, Propranolol
Costa et al., 2018^13^	Prospective cohort study	To evaluate the use of off-label and unlicensed medicines in a NICU of a maternity teaching hospital specialized in high-risk pregnancy	NICU: Spironolactone, Furosemide, and Hydrochlorothiazide
Britto et al., 2017^26^	Descriptive study	To describe the use profile of pediatric magistral formulations in a public maternal and child hospital, reference in southwest Bahia (Brazil).	P+N: Captopril, Spironolactone, Hydrochlorothiazide
Gonçalves et al., 2017^27^	Prospective study	To investigate the use of off-label drugs in NICUs according to ANVISA and the FDA	NICU: Amiodarone, Captopril, Carvedilol, Spironolactone, Furosemide, Hydrochlorothiazide, Nifedipine
Pereira et al., 2016^28^	Descriptive study	To study the use of magisterial oral solutions and suspensions in newborns and children at a university hospital	P+N: Amiodarone, Captopril, Enalapril, Spironolactone, Furosemide, Hydralazine, Hydrochlorothiazide, Propranolol
Souza Junior et al., 2016^29^	Retrospective cohort study	To describe the drugs prescribed for different groups of neonates admitted to the NICU and to analyze the off-label use and the harmful potential of the drugs	NICU: Spironolactone, Captopril, Furosemide, Propranolol, Epinephrine (the type of transformation is not mentioned in the study)
Dos Santos et al., 2012^30^	Cross-sectional study	To describe and determine the extent of use of unlicensed, off-label, and high-risk drugs in the general pediatric units of a university hospital in southern Brazil	P+N: Spironolactone, Furosemide
Ferreira et al., 2012^31^	Cross-sectional study	To describe the use and determine the prescription prevalence of off-label and unlicensed medicines in a pediatric intensive care unit of a hospital in southeastern Brazil	P+N: Captopril, Furosemide, Hydrochlorothiazide
Costa et al., 2009^3^	Descriptive study	To identify drugs whose dosage form or formulation represents a problem in pediatrics (problem medication)	P+N: Captopril, Digoxin, Spironolactone, Furosemide, Hydrochlorothiazide, Nifedipine
Costa et al., 2009^32^	Descriptive study	To identify drugs that present difficulties for their pediatric use in Brazil	P+N: Captopril, Spironolactone, Hydrochlorothiazide, Propranolol

P: pediatrics; N: neonatology; NICU: Neonatal Intensive Care Unit; ANVISA: Brazilian Health Regulatory Agency; FDA: U.S. Food and Drug Administration.

**Figure 2:**
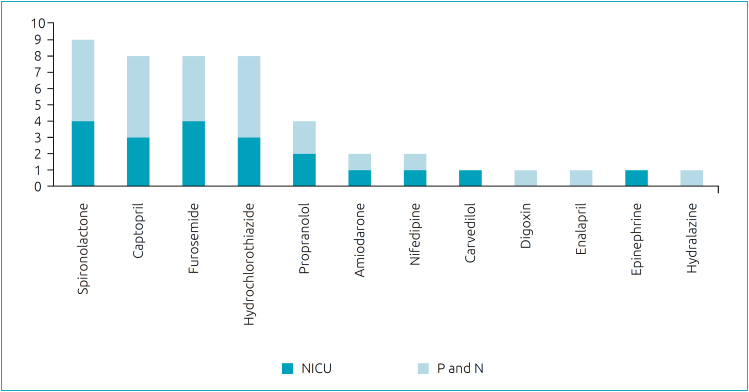
Frequency of mentions of cardiovascular medicines in the sample of studied articles.

For better data analysis, the cardiovascular drugs indicated in the studies are presented in Figure 2, according to the frequency in which they were mentioned and the type of hospitalization unit in which they were mentioned. The 10 studies considered in this review mentioned the most indicated cardiovascular medicines in pediatrics and neonatology. Among them, in descending order, are Spironolactone,^13,25–30^ Captopril,^25,29–31^ Furosemide,^13,25–31^ Hydrochlorothiazide,^13,25–28,31^ Propranolol,^25–29^ Amiodarone,^27,28^ Nifedipine,^27^ Carvedilol,^27^ Digoxin,^3^ Enalapril,^32^ Epinephrine,^29^ and Hydralazine.^28^

The present study also compared the presence of the cardiovascular drugs mentioned in this review with the List of Essential Medicines for Children, of the World Health Organization,^22^ and verified the existence of registration of the oral liquid dosage form in Brazil and in the United Kingdom, as described in Table 2. It was found that none of the mentioned cardiovascular medicines has a registration of the oral liquid dosage form in Brazil, and only four drugs (Spironolactone, Furosemide, Captopril, and Propranolol) have a registration of the oral liquid dosage form in the United Kingdom.

**Table 2:** Characteristics of registrations of oral liquid dosage forms of cardiovascular medicines important in neonatology in Brazil and in the United Kingdom.

Medicines	ATC code	Registration of the oral liquid dosage form in the United Kingdom	Registration of the oral liquid dosage form in Brazil
Spironolactone	C03DA01	Yes	No
Hydrochlorothiazide	C03AA03	No	No
Furosemide	C03CA01	Yes	No
Captopril	C09AA01	Yes	No
Propranolol	C07AA05	Yes	No
Enalapril	C09AA02	No	No
Carvedilol	C07AG02	No	No
Hydralazine	C02DB02	No	No

## DISCUSSION

The studies used in this review state that the most indicated cardiovascular medications in pediatrics and neonatology are: Spironolactone,^13,25–30^ Captopril,^25,29–31^ Furosemide,^13,25^ Hydrochlorothiazide,^13,25–28,31^ Propranolol,^25–29^ Amiodarone,^27–28^ Nifedipine,^27^ Carvedilol,^27^ Digoxin,^3^ Enalapril,^28^ Epinephrine,^29^ and Hydralazine.^28^ Considering that the articles have representative data of the routine of that clinical unit, the most cited drugs are more likely to be part of the list of medicines standardized for a given establishment. Therefore, these drugs are important medicines in neonatology.

Costa et al.^3^ evaluated the demand for compounding oral liquid pharmaceutical formulations containing cardiovascular drugs, through interviews with pediatricians. In the interview, physicians identified the main drugs whose pharmaceutical formulation represented a problem in pediatrics, such as Captopril (5mg/mL), Spironolactone (5mg/5mL, 10mg/5mL, 25g/5mL, 50mg/5mL, 100mg/5mL), Hydrochlorothiazide (50mg/5mL), and Propranolol (4 and 8mg/mL).

Ferreira et al.^31^ described the cardiovascular medications prescribed to different groups of neonates through a cross-sectional study carried out in the Pediatric Intensive Care Unit (ICU) of a hospital in the city of Belo Horizonte (state of Minas Gerais, Brazil), and found prescriptions containing the drugs Captopril, Furosemide, and Hydrochlorothiazide for neonatal use. Pereira et al.^28^ studied the use of magistral oral solutions and suspensions in newborns and children at a university hospital, by analyzing compounding request forms for patients admitted to the neonatal, obstetrics, and pediatrics ICU. This study mentioned pediatric oral liquid preparations of Captopril, Furosemide, Spironolactone, Hydrochlorothiazide, Hydralazine, Amiodarone, Propranolol, and Enalapril, which were produced in the compounding pharmacy, under the responsibility and guidance of the pharmacist.

Passos et al..^25^ studied prescriptions for newborn patients with a mean age of 15 days and analyzed the prescriptions of compounded medication for newborn patients and their pharmacotechnical feasibility. The authors observed that drugs were obtained in the form of solutions based on the drug Captopril; as suspensions, for delivering the drugs Spironolactone and Furosemide; and as syrup, for delivering the drug Propranolol.

Such drugs are not marketed in Brazil in liquid form, but are used in hospitals to make treatments viable. Therefore, they are invariably prepared extemporaneously.^25,31^ They are obtained from adaptations of dosage forms, through the transformation of capsules or tablets into liquid formulations such as solution, suspension, or syrup. This process occurs in 75.63% of medications in the pediatric ICU, due to patients’ difficulty or inability to swallow and the need for lower doses.^26–28^

A relevant aspect in relation to obtaining pediatric medicines is the dose adjustment, which must be performed through complex calculations, which in turn are necessary to adapt the formulation indicated for adult use to another formulation aimed at pediatric use, usually with very small doses, as those used for neonates. Thus, Ferreira et al.^31^ found that the cardiovascular drugs most prescribed in underdoses were Spironolactone, Captopril, and Furosemide, while Captopril and Digoxin were among the most prescribed drugs in overdoses, with the majority of overdoses occurring among lactating women.

Within this scenario, it is necessary to understand that the production of extemporaneous formulations, from the transformation of solid oral dosage forms into liquid oral dosage forms, represents a risk due to the presence of different excipients in the composition of these pharmaceutical products, which can cause adverse events in patients. In addition, there is the lack of information on bioavailability and physical, chemical, and microbiological stability of the resulting formulations that are not readily available,^16^ which are essential to adapt the shelf life, packaging, storage, control procedures of quality, and traceability of these drugs.^28^ In this context, the use of such medications has become less safe and with less predictable results.

Moreover, the non-complete use of the compounded medication by the pediatric patient can occur and generate financial losses. Nevertheless, given the total absence of a suitable dosage form for this group of patients, compounding can still represent one of the alternatives to make the prescribed treatment feasible.^27–30^ Thus, the compounding of medicines has a fundamental role in obtaining the feasibility of the drug therapy in neonatology.

Regarding the presence of the cardiovascular drugs mentioned in this review that are part of the List of Essential Medicines for Children of the World Health Organization,^26^ it was verified that, with the exception of Captopril and the liquid oral form of Propranolol, the main drugs, such as Spironolactone, Furosemide, and Hydrochlorothiazide, are referenced for the treatment of priority diseases. Furosemide is included in the core list of the document, which deals with the minimum drugs necessary for an effective, safe, and cost-effective treatment for priority diseases in the health system. Conversely, Spironolactone and Hydrochlorothiazide, despite being part of the document, are included in the complementary list, concerning medicines to treat priority diseases that require specific diagnosis and therapy with a specialized professional, and must be evaluated as for safety and cost-effectiveness.

Captopril, although not listed in the document of the World Health Organization, has a well-established use for the treatment of various conditions in neonatology, such as: hypertension, heart failure, proteinuria in nephritis, and diabetic nephropathy in type 1 diabetes mellitus. The protocols for use, indication, forms of administration, and monitoring care are described for term and preterm neonates in the BNFC.^21^

Digoxin, despite being part of the core list of essential medicines for children of the World Health Organization and having its use established by the BNFC, was not observed in the NICU in this review. This result can be attributed to the fact that there are drugs with a higher therapeutic index, such as Propranolol and Amiodarone, to treat supraventricular arrhythmias, which is the main indication for the use of Digoxin in neonatology.^21^

The therapeutic class of diuretics includes the main prescribed drugs, as they are medicines of first choice in the treatment of pulmonary edema in neonates, which can be caused by conditions such as pulmonary stress or bronchopulmonary dysplasia, as well as in the clinical management of neonates with congenital heart disease. Overall, diuretics can be prescribed together, as long as they belong to different classes and act synergistically: Spironolactone as a potassium-sparing diuretic; Hydrochlorothiazideas a thiazide diuretic; and Furosemide as a loop diuretic.^21^

In addition, it was verified that, among the main cardiovascular medicines compounded for neonatology in Brazil, none has a registration of the oral liquid dosage form at ANVISA. Conversely, in the United Kingdom, four are registered, one of them being Chlorothiazide (ATC code: C03AA04), a thiazide diuretic that replaces Hydrochlorothiazide. Chlorothiazide is registered as a liquid oral dosage form and its use is licensed for neonatology.^21^ This can be attributed to the fact that there are regulatory incentives to gather information for important medicines in pediatrics, such as, for example, the pediatric investigation plan, in the registration of a particular drug or class of drugs, in addition to financial incentives and patent extension for companies that produce drugs with information on efficacy and safety for pediatric use.^2,4^

None of the cardiovascular medicines compounded for neonatology had information on their use in the latest edition of the National Therapeutic Form.^24^ In comparison with the BNFC, it can be noted that there is no official reference of information for the safe and effective use of important drugs in neonatology (and pediatrics as a whole) in Brazil. The BNFC has pieces of information ranging from protocols for the administration and maintenance of therapy, standardized strategies for compounding extemporaneous forms, and data on efficacy and safety, including management of adverse events, doses, and medications registered and available on the market, and charged costs.

All cardiovascular drugs described in this review, regarding the demand for pharmaceutical compounding, can be classified as compounded medications necessary in neonatology, due to the lack of registration of the oral liquid dosage form in Brazil. This approach takes into account the adaptability of oral liquid dosage forms with regard to their cost-effectiveness and the suitability of this form concerning the anatomical and physiological immaturity of the neonate. This approach has already been used by Castro et al.,^33^ and the authors highlighted the need to also evaluate data on toxicity of excipients to ensure that the formulation is suitable for this age group.

Furthermore, there are few governmental, regulatory, or financial incentives for the manufacture and/or registration of dosage forms intended for pediatrics in the country, or for gathering safety and efficacy information, or even an official reference for unified information, for cases in which the need for compounding and the use of the unlicensed form are justified by the principle of beneficence. However, in 2018, a document was published within the scope of the National Medicines Policy entitled *Assistência farmacêutica em pediatria no Brasil* (Pharmaceutical assistance in pediatrics in Brazil),^18^ which compiles recommendations and strategies that address these aforementioned points, seeking, in the long term, to correct the gap in access to safe and effective medications and to ensure the rational use of medicines for the pediatric population.

Finally, it is worth highlighting that the use of off-label and unlicensed drugs in neonatal and pediatric patients may be associated with the emergence of adverse events.^11^ Carvalho et al.^16^ correlated the non-standardized and unlicensed use of drugs in the NICU with severity scores and showed that the greater the severity of the clinical involvement, the greater the chance of the patient receiving a drug of this classification.

The main limitation of this study concerns the search for Brazilian articles, which should be considered regarding the generalization of data, despite the comparison with the BNFC.

In this study, through the literature review, it was possible to map the cardiovascular drugs needed in neonatology in Brazil, such as Spironolactone, Captopril, Furosemide, Hydrochlorothiazide, Propranolol, Amiodarone, Nifedipine, Carvedilol, Digoxin, Enalapril, Epinephrine, and Hydralazine. Despite their well-reported use in the literature, these medications are not registered as an oral liquid dosage form in the country, in contrast to the United Kingdom. Moreover, none of these drugs has an official reference for safe and effective use in neonatal patients in Brazil.

The lack of appropriate formulations for pediatric use has repercussions on medical practice in Brazil and is exacerbated by the lack of adequate conditions for the compounding of medications by pharmacists in Brazilian hospitals. Such factors highlight the need for investment in the production and registration of medicines suitable for the pediatric population with the creation of regulatory and financial incentives for the pharmaceutical industry, as well as validation, standardization, and provision of information on methods for compounding extemporaneous formulations in the neonatology to increase the safety of these drugs, which are essential to improve the access of this group, therapeutic orphan, to safe, effective, and quality treatment and to reduce health inequities.
